# The impact of communication skills training on patient-centered attitude and patient satisfaction: A longitudinal study of Egyptian physicians

**DOI:** 10.1016/j.pmedr.2025.103117

**Published:** 2025-05-28

**Authors:** Mahmoud M. Samir, Nehal Mohamed Eisa, Heba Khaled, Wajid Syed, Mahmood Basil A. Al-Rawi, Ahmed Essam Abou Warda, Nourhan M. Kamal, Reem S. Mahmoud, Abdelrahman SH. Refaee

**Affiliations:** a ICH-CRC, MOHP, Giza 12585, Egypt; b Clinical Research Department at Giza Health Affairs Directorate, MOHP, Giza 12585, Egypt; c Clinical Pharmacy Department, Faculty of Pharmacy, October 6 University, Giza 12585, Egypt; d Department of Biochemistry, Faculty of Pharmacy, Cairo University, Cairo, Egypt; e Department of Clinical Pharmacy, College of Pharmacy, King Saud University, Riyadh 11451, Saudi Arabia; f Department of Optometry, College of Applied Medical Sciences, King Saud University, Riyadh 11451, Saudi Arabia; g Department of Pharmacotherapy and Translational Research, College of Pharmacy, University of Florida, USA

**Keywords:** Patient-centered care, Communication skills training, Doctor-patient relationship, Patient satisfaction, Egypt

## Abstract

**Objective:**

Patient-centered care (PCC) provides recognized global benefits but remains underexplored in the Middle East and North Africa (MENA) region, particularly in Egypt. This study assesses the impact of communication skills training on doctor-patient relationships, patient-centered attitudes, and patient satisfaction in healthcare centers across Giza governorate.

**Methods:**

From November 2022 to January 2024, a multi-center longitudinal study was carried out in healthcare centers across the Giza governorate in Egypt, involving 102 physicians and 257 patients. The physicians completed a two-day training program titled “Effective Communication Skills Training,” based on the principles of patient-centered care. To assess outcomes, the Patient-Practitioner Orientation Scale (PPOS) was used to measure physicians' attitudes before and after the training, while the Patient-Doctor Relationship Questionnaire (PDRQ-9) evaluated patient satisfaction.

**Results:**

Pre- and post-training PPOS scores exhibited a slight increase (mean difference = 0.06), with significant improvements for participants with pre-training scores ≤3 (*p* < 0.001). No overall significant difference was noted between pre- and post-training scores. In the sharing subdomain, surgical specialty and pre-training sharing scores were significant predictors of PPOS improvement. Patient satisfaction scores rose post-training, with a mean score of 4.44 ± 1 compared to 4.22 ± 0.67 pre-training (*p* = 0.001).

**Conclusion:**

The study demonstrates that communication skills training based on the PCC model effectively enhances PCC and patient satisfaction among physicians, indicating that targeted training can address PCC knowledge gaps and improve patient care.

## 1 Introduction

The emphasis on patient-centered care (PCC) has gained significant attention from policymakers globally. Despite extensive evidence on the benefits and challenges of PCC in Western countries, the application and endorsement of PCC appear somewhat limited in the Middle East and North Africa (MENA) region. Also, there has been a lack of comprehensive research on its practice in the MENA region, including Egypt (Alkhaibari et al., 2023).

PCC has been linked to increased patient satisfaction (Fossum and Arborelius, 2004), better patient adherence (Zolnierek and Dimatteo, 2009), better patient health outcomes, such as decreased anxiety and discomfort; and improved self-reported health (Stewart et al., 2007), enhanced physiological state (Rao et al., 2007); and decreased healthcare costs (Stewart et al., 2011).

Although PCC lacks a standardized definition, the Institute of Medicine (IOM) outlines eight key sub-dimensions for delivering PCC: (1) respecting patient preferences, (2) providing information and education, (3) ensuring access to care, (4) offering emotional support, (5) involving family and friends, (6) ensuring continuity and transitions, (7) maintaining physical comfort, and (8) coordinating care (Hong and Moliterno, 2020).

Educational initiatives aimed at promoting PCC are frequently discussed in medical education literature. Training programs for medical professionals have been demonstrated to enhance empathy and PCC, as evidenced by various assessment tools (King and Hoppe, 2013).

Given the unique cultural and healthcare contexts in Egypt and other MENA countries, it is crucial to conduct more investigations and research to understand how PCC can be effectively implemented and adapted to meet the specific needs of patients in this region (Alkhaibari et al., 2023).

Patient satisfaction is crucial for all stakeholders and is widely used to evaluate health services. It increases adherence to treatment (Sánchez-Piedra et al., 2014), boosts staff satisfaction and reduces malpractice for physicians (Welch, 2010), and helps governments identify areas for improvement (Sánchez-Piedra et al., 2014). Overall, it serves as a key indicator of care quality (Fenton et al., 2012).

Two local studies on the doctor-patient relationship and patient satisfaction in Egyptian healthcare centers found total satisfaction to be below acceptable levels. They recommended regular communication skills training for physicians and other medical professionals (Farghaly et al., 2021; Hegazy et al., 2021).

This study aims to evaluate the impact of effective communication skills training based on the PCC model on doctor-patient relationship (DPR), patient-centered attitude, and patient satisfaction in Giza governorate healthcare centers.

## 2 Methods

### 2.1 Study design and participants

This quasi-experimental, multi-center longitudinal study was carried out from November 2022 to January 2024 at healthcare centers in the Giza governorate, one of Egypt's largest healthcare networks. The study included 102 physicians and 257 patients. Physicians were divided into two groups: 51 received communication skills training based on the Patient-Centered Care (PCC) model of Patient-Doctor Relationship (PDR), while the remaining 51 formed the control group. Patient satisfaction was measured as a primary health outcome.

Eligible physicians were those practicing at selected healthcare facilities, with at least six months of experience and willing to provide consent. Exclusions included individuals in administrative roles, those on short-term rotations, and those who had received formal communication training within the last year. Eligible patients were adults (≥18 years), cognitively and physically capable of participating, and provided consent. Patients with severe psychiatric or cognitive impairments or critical illnesses were excluded.

Physicians were invited to participate through routine administrative channels as part of the standard training programs in Giza hospitals. The training sessions were scheduled during working hours to ensure minimal disruption to clinical duties. Participation was voluntary, with certification provided as the sole incentive. Since the training was part of the ongoing professional development framework, individual invitations and formal response rates were not tracked. All participating physicians were hospital-based, providing care for both inpatients and outpatients, thus representing a diverse patient population.

### 2.2 Training intervention

The “Effective Communication Skills Training” is a two-day program designed to improve healthcare professionals' communication skills. Delivered by two facilitators, the course includes eight hours of interactive sessions. Day one covers the basics of the doctor-patient relationship and essential communication skills, featuring theoretical insights and practical exercises. Day two focuses on PCC and patient satisfaction, explores the eight key sub-dimensions for delivering PCC (Hong and Moliterno, 2020), methods for integrating patient preferences into clinical practice, techniques for eliciting and addressing patient concerns, and the evaluation of patient satisfaction metrics. The training combines presentations, discussions, and interactive activities to foster empathetic, effective communication in healthcare settings. 20 to 24 physicians participated in each training group.

### 2.3 Study measures

All physicians and patients underwent an assessment of socio-demographic characteristics. To evaluate physicians' personal beliefs and preferences regarding patient-centered care (PCC) in doctor-patient relationships, we utilized the Patient-Practitioner Orientation Scale (PPOS), developed by Krupat et al. (2001). The PPOS is an 18-item instrument designed to assess attitudes toward patient-centeredness in healthcare settings. Each item is rated on a six-point Likert scale, ranging from “Strongly Disagree” to “Strongly Agree,” with higher scores indicating a more patient-centered orientation. The scale comprises two subscales: “Sharing,” which evaluates beliefs about the distribution of power and information between patient and practitioner, and “Caring,” which assesses the importance placed on understanding the patient's psychosocial context. Higher scores reflect a greater patient-centered orientation rather than a practitioner-centered one. The PPOS has been validated in various settings, with reported internal consistency (Cronbach's alpha) ranging from 0.73 to 0.88 (Trapp and Stern, 2013).

To measure patient satisfaction with healthcare services, we employed the Patient-Doctor Relationship Questionnaire (PDRQ-9), developed by van der Feltz-Cornelis et al. (2004). The PDRQ-9 is a validated nine-item instrument that assesses the quality of the doctor-patient relationship from the patient's perspective using a nine-point Likert scale. It has demonstrated high internal consistency, with Cronbach's alpha ranging from 0.94 to 0.95, and strong construct validity in primary care research.

Physicians were assessed at baseline and one week after completing the communication skills training course. Patient satisfaction was evaluated after consultations with physicians who had participated in the training. Both scales are widely used and psychometrically robust for evaluating communication and patient satisfaction in healthcare settings.

### 2.4 Ethical consideration

All procedures involved in the present study were carried out in conjunction with good clinical practice, the Declaration of Helsinki, and World Health Organization Guidelines. The “Research Ethics Committee” in the Central Directorate for Research and Health Development in MOHP (REC No. 16-2022/14) reviewed and accepted the research protocol. Written informed consent was obtained from each participant.

### 2.5 Statistical methods

The sample size was determined using OpenEpi, Version 3, with a power of 80 % and α = 5 %. To detect a meaningful difference, 74 physicians (37 per group) and 198 patients (99 per group) were required. Allowing for a 20 % dropout rate, we aimed to recruit 88 physicians and 238 patients.

The overall PPOS score was computed as the unweighted mean of all 18 items. Furthermore, the Caring and Sharing sub-scores were assessed independently, with each sub-scale comprising nine items. To assess the normality of the pre-training total score (T1), post-training total score (T2), and their difference (T2 - T1), we employed the Kolmogorov-Smirnov test. A *p*-value greater than 0.05 indicates that the data do not significantly deviate from a normal distribution. The caring and sharing sub-scores were calculated as unweighted means, with higher scores reflecting a more positive attitude toward care and sharing, respectively.

To determine a data-driven cutoff for subgroup analysis, we used ROC curve analysis to identify the pre-training PPOS score that best distinguishes between individuals who showed a positive absolute difference (improvement) in post-training scores and those who showed no change or a negative difference. This approach ensures that the chosen threshold effectively differentiates between these groups based on sensitivity and specificity.

A multivariate linear regression analysis was conducted to evaluate the relationship between baseline PPOS scores and key demographic variables, including gender, specialty, and years since graduation. Additionally, a separate multivariate linear regression was performed to investigate the relationship between the change in PPOS score, as well as changes in sharing and caring scores, with the following explanatory variables: gender, specialty, years of practice, and pre-training score. Data were assessed for potential issues that could violate the assumptions of linear regression. Multicollinearity was checked using the Tolerance and Variance Inflation Factor (VIF). The independence of observations was evaluated using the Durbin-Watson test. Heteroskedasticity and normality of residuals were assessed using the Breusch-Pagan or White's test and the Shapiro-Wilk test, respectively. A *p*-value <0.05 was considered statistically significant. When necessary, the HC3 method correction was applied to address heteroskedasticity, ensuring the validity of the regression results.

for the patient satisfaction score survey, univariate analysis of categorical variables was performed using the Chi-square test, while Continuous variables were analyzed using either parametric (independent samples *t*-test) or non-parametric (Mann-Whitney *U* test) approach, depending on whether the data were normally distributed as determined by the Kolmogorov–Smirnov test.

The statistical analysis was conducted using SPSS version 27.0 (SPSS Inc., Chicago, III, USA).

## 3 Results

### 3.1 Baseline characteristics of participating physicians

Table 1 shows the characteristics of the participating doctors. The study involved 102 doctors. Most of the participants were women (*n* = 75, 73.5 %), with men representing a smaller proportion (*n* = 27, 26.5 %). Over half of the doctors studied were in surgical specialties (*n* = 56, 54.9 %), while the rest (*n* = 46, 45.1 %) specialized in nonsurgical/internist medicine. The majority of physicians had been practicing medicine for more than 10 years (*n* = 83, 81.4 %), while a smaller proportion had been practicing for less than 10 years (*n* = 19, 18.6 %).

**Table 1:** Demographic and professional characteristics of physicians participating in communication training program in Giza Governorate, Egypt (November 2022–January 2024) (*N* = 102).

Variable	Category	Frequency (n)	Percentage (%)
Gender	Female	75	73.5
	Male	27	26.5
Specialty	Non-surgical/internal	46	45.1
	Surgical	56	54.9
Years of practice	Less than 10 years	19	18.6
	10 years or more	83	81.4

### 3.2 Associations between participants characteristics and baseline PPOS scores

Multivariate linear regression analysis revealed that none of the demographic variables examined were significant predictors of baseline Patient-Practitioner Orientation Scale (PPOS) scores. Specifically, participants' specialty was not significantly associated with baseline PPOS scores (β = −0.08, 95 % CI −0.34 to 0.17, *p* = 0.519). Likewise, years since graduation did not significantly predict baseline scores (β = 0.0, 95 % CI −0.02 to 0.01, *p* = 0.550). Although male participants showed a non-significant trend toward higher baseline scores (β = 0.24, 95 % CI −0.05 to 0.54, *p* = 0.100), this finding did not reach statistical significance. Overall, the results suggest that gender, specialty, and experience were not associated with patient-centred attitudes at baseline as measured by the PPOS. These results are presented in Table 2.

**Table 2:** Factors associated with changes in patient-centered attitudes among physicians following communication training in Giza Governorate, Egypt (November 2022–January 2024).

Outcome	Predictor	B Coefficient	95 % Confidence Interval
Delta PPOS	Sex (male)	0.034	(−0.154, 0.223)
	Specialty (surgical)	−0.101	(−0.267, 0.064)
	Years of practice (<10 years)	0.009	(−0.184, 0.203)
	Pretraining PPOS score T1	−0.461	(−0.588, −0.334)
Delta Sharing^a^	Sex (male)	0.184	(−0.055, 0.422)
	Specialty (surgical)	−0.219	(−0.420, −0.019)
	Years of practice (<10 years)	−0.033	(−0.270, 0.204)
	Pretraining sharing score	−0.38	(−0.621, −0.138)
Delta Caring	Sex (male)	−0.122	(−0.371, 0.127)
	Specialty (surgical)	0.008	(−0.209, 0.226)
	Years of practice (<10 years)	0.065	(−0.190, 0.320)
	Pretraining caring score	−0.5	(−0.658, −0.343)

### 3.3 Comparison of pre-training and post-training Patient-Practitioner Orientation Scale (PPOS) scores

The analysis of total PPOS scores revealed a small overall increase following training. The mean pre-training score (T1) was 3.16 (SD = 0.598), while the mean post-training score (T2) was 3.22 (SD = 0.487), with a mean difference (T2 − T1) of 0.06 (SD = 0.463). Further subgroup analysis showed that participants with lower baseline scores (T1 ≤ 3) experienced a statistically significant improvement after the training, with mean pre-training and post-training scores of 2.67 ± 0.32 and 2.94 ± 0.42, respectively (*p* < 0.001). In contrast, participants with higher pre-training scores (T1 > 3) did not show a significant change post-training (*p* > 0.05). These comparisons were based on paired score analysis and are not shown in Table 2, which instead focuses on predictors of change.

### 3.4 Factors influencing differences in overall and subdomain (sharing and caring) PPOS scores

Table 2 presents the results of multivariate linear regression models examining predictors of change in overall PPOS scores and its two subdomains: sharing and caring. The strongest predictor of change in overall PPOS score was the baseline (pre-training) score itself. Higher pre-training scores were significantly associated with smaller improvements following training (β = −0.46, 95 % CI -0.588 to −0.334, *p* < 0.001). Other variables—including gender, specialty, and years of practice—were not significant predictors of change in the total PPOS score.

For the sharing subdomain, surgical specialty was a significant negative predictor of improvement, with participants from surgical specialties showing smaller gains compared to their non-surgical counterparts (β = −0.219, 95 % CI -0.420 to −0.019, *p* = 0.032). Similarly, higher baseline sharing scores were associated with smaller improvements post-training (β = −0.380, 95 % CI -0.621 to −0.138, *p* = 0.002). In the caring subdomain, only the baseline caring score significantly predicted change, with higher pre-training values associated with smaller improvements (β = −0.500, 95 % CI -0.658 to −0.343, p < 0.001). Other demographic variables did not significantly influence changes in either subdomain.

### 3.5 Patient satisfaction measurements

The study included a total of 257 patients, with 128 in the pre-training group and 129 in the post-training group. The mean age of participants was 36.5 ± 21 in the pre-training group and 37.22 ± 11.74 in the post-training group. There was no significant difference in age between the two groups (*p* = 0.98). Regarding sex distribution, the pre-training group included 78 (60.9 %) females and 50 (39.1 %) males, while the post-training group included 83 (64.3 %) females and 46 (35.7 %) males. No significant difference was observed in sex distribution between the groups (*p* = 0.57), indicating they were matched in terms of age and sex. The mean patient satisfaction score (measured by PDRQ-9) was 4.22 ± 0.67 in the pre-training group and 4.44 ± 1 in the post-training group. A statistically significant difference in satisfaction scores was found between the two groups (*p* = 0.001*), with higher satisfaction reported in the post-training group. All results are shown in Table 3.

**Table 3:** Comparison of Demographics and Patient Satisfaction (PDRQ-9) scores before and after physician communication training in Giza Governorate, Egypt (November 2022–January 2024).

Variable	Pre-training (*n* = 128)	Post-training (*n* = 129)	P-value
Patient Age (Mean ± SD)	36.5 ± 21	37.2 ± 11.7	0.98
*Sex, n (%)*			
Female	78 (60.9)	83 (64.3)	0.57
Male	50 (39.1)	46 (35.7)	
Score (Mean ± SD)	4.22 ± 0.67	4.44 ± 1	0.001

## 4 Discussion

Comprehensive analysis of outcomes of the effective communication skills training tailored for physicians has yielded insightful and noteworthy results. The mean PPOS score in our study ranged from 3.16 before training to 3.22 after training. Comparisons with studies from other countries indicate notable variations in PPOS scores. Research in Spain reported a PPOS score of 3.75 (Perestelo-Pérez et al., 2021), while studies in Saudi Arabia and Sudan recorded scores of 3.40 (Mohamed et al., 2024) and 3.75 (Mohamed et al., 2019), respectively. Outside the Middle East and North Africa, our PPOS score remains lower than those reported in Mali (3.38) (Hurley et al., 2018), Pakistan (3.40) (Ahmad et al., 2015), China (3.63) (Liu et al., 2019), and Brazil (4.57) (Barbato et al., 2023).

These variations highlight the impact of cultural and systemic factors on patient-centered attitudes. Our study adds to this global perspective, underscoring the importance of tailored strategies to enhance patient-centered care within the Egyptian healthcare system.

Detailed analysis of the pre- and post-training PPOS scores revealed that physicians who entered the training with lower baseline PCC knowledge, as evidenced by their pretest PPOS scores, experienced the most pronounced improvements post-training. Multiple statistical analysis methods confirmed that this group exhibited significant gains in their post-test performance, suggesting that the training methodology is particularly effective in elevating the competencies of participants with initially lower patient-centeredness levels. This finding underscores the potential of targeted educational interventions to bridge PCC knowledge gaps in medical practice.

Notably, the greatest improvement in PPOS scores was observed among physicians with lower baseline patient-centeredness, highlighting the training's ability to bridge knowledge gaps and enhance PCC where it is most needed. This finding underscores the potential for targeted interventions to drive meaningful behavioral change, even in those initially less inclined toward patient-centered care.

This was in agreement with a study by Lim et al. (2023), where a brief, single-session training led to observable enhancements in the total PPOS among physicians. Initially, there was a stronger inclination toward the caring rather than the sharing subscale, though the training notably increased the latter. This was in line with multiple research findings reviewed by Alkhaibari et al. (2023) showing that physicians who have received training in communication with patients typically achieve higher scores in patient-centeredness, both overall and in the two subscales of the PPOS. Similarly, communication skills training programs for physicians enhanced PDR and PCC, using different assessment tools (Epstein et al., 2017; Du et al., 2022).

The relationships between physicians' demographic and professional variables and their patient-centered attitudes were examined in different studies. Studies indicate that female physicians generally exhibit more patient-centered attitudes (using PPOS) compared to male physicians, and non-surgical physicians score higher on patient-centeredness than surgeons (Liang et al., 2023; Chan and Ahmad, 2012). Additionally, longitudinal research shows a significant decline in patient-centered attitudes during residency, with male residents exhibiting a greater decline in caring attitudes (Ishikawa et al., 2018).

Our findings align with these observations, as female physicians in our study demonstrated higher PPOS scores than their male counterparts. This may be attributed to differences in communication styles, as previous research suggests that female physicians tend to engage in more participatory and empathetic interactions, allocate more time to patient communication, and place a stronger emphasis on psychosocial aspects of care. These factors contribute to a more patient-centered approach, as reflected in their higher PPOS scores.

Similarly, non-surgical physicians in our study exhibited greater patient-centeredness compared to surgeons. This difference is likely influenced by the nature of their clinical practice. Non-surgical specialties often involve chronic disease management and long-term patient relationships, necessitating a stronger emphasis on communication, shared decision-making, and ongoing patient engagement. In contrast, surgical specialties are more procedure-focused, with interactions that often prioritize efficiency and technical precision over extended patient dialogue. These factors may explain why non-surgical physicians tend to have higher patient-centeredness scores.

Despite these trends, our regression model showed that gender, specialty, and years of practice category were not significant predictors of the change in PPOS scores post-training. This suggests that while baseline differences exist, the training program was equally effective across all demographic and professional groups. The uniform impact of the training underscores its broad applicability and effectiveness in enhancing patient-centered communication, regardless of physicians' background or specialty.

We tried to assess these demographic and professional variables (such as gender, years of practice, and medical specialty) as predictors of the difference in the overall PPOS score and its sub-domains (sharing and caring). Our regression model showed that gender, specialty, and years of practice category were not significant predictors of the difference. This lack of variance implies that the training program's efficacy is uniformly distributed, regardless of a physician's demographic background or professional experience. It suggests a universal applicability and effectiveness of the training content, making it a versatile tool for continuing medical education.

These outcomes highlight the effective communication skills training's robustness in enhancing physician PCC knowledge and skills across a diverse cohort. The substantial improvement observed in those with lower initial PPOS scores is particularly encouraging, indicating the potential for significant professional development through targeted educational programs. Moreover, the uniform effectiveness across various demographics and specialties speaks to the broad applicability of the training, ensuring that it benefits a wide range of practitioners.

The study evaluated patient satisfaction (using PDRQ9) after consultations with physicians who underwent effective communication skills training. It included participants divided into pre-training and post-training groups. Both groups were matched in age and sex distribution. The post-training group showed significantly higher patient satisfaction compared to the pre-training group.

Research on the impact of communication skills training on patient satisfaction has shown mixed results with different sets of assessment tools. Stein et al. (2005) found significant improvement in outpatient satisfaction after a five-day course. Similarly, Boissy et al. (2016) reported higher patient satisfaction following an eight-hour relationship-centered communication training at the Cleveland Clinic. This is further supported by the review by Drossman et al. (2021), which confirms that training in communication skills has been shown to increase patient satisfaction and improve outcomes. However, Curtis et al. (2013) found no improvement in patient and family-reported outcomes following a communication skills intervention for physicians.

The significant increase in patient satisfaction post-training highlights the practical impact of communication skills development. Higher patient satisfaction scores suggest that the training translated into more effective physician-patient interactions, aligning with existing literature on the link between communication quality and patient experience.

Our study has several significant implications. It is the first to evaluate a patient-centered communication skills training program for all physicians in a large multispecialty setting in Egypt, with an emphasis on patient satisfaction.

This study's findings demonstrate that a brief and practical communication skills training program grounded in the PCC model training can effectively improve physicians' readiness for patient-centered care and enhance patient satisfaction. This approach could serve as a model for similar initiatives elsewhere. Expanding such models could lead to notable improvements in communication quality and patient relationships in modern medical practice. However, further research with larger samples and extended follow-up is needed to determine whether the benefits of the training are sustained over the long term and applicable to other settings.

## 5 Strengths and limitations

This study's strength lies in its pioneering approach to enhancing communication skills for physicians in Egypt, anchored in the principles of patient-centered care (PCC). By focusing on a tailored training program, it provides valuable insights into how systematic, targeted interventions can foster meaningful improvements in physician-patient interactions. The study stands out for its broad applicability, demonstrating that the training is effective across a diverse range of physicians, regardless of gender, specialty, or experience. Moreover, its emphasis on patient satisfaction ties the impact of improved communication directly to better patient outcomes, setting a precedent for future educational initiatives in healthcare. As the first comprehensive study of its kind in Egypt, it offers a promising model for advancing PCC both locally and globally.

This study has some limitations. Firstly, patients interacted with multiple providers across departments, making it difficult to attribute satisfaction scores to individual physicians or track specific PPOS changes. Secondly, although we matched pre- and post-training patient groups by age and sex, they were not the same individuals, which limits longitudinal analysis. Additionally, clustering effects were not applicable, as patients were not assigned to specific physicians, making a mixed-effects model unnecessary. Finally, while improvements in patient-centeredness and satisfaction were observed, future studies with controlled patient-physician assignments are needed to explore individual-level effects and long-term outcomes.

## 6 Conclusion

This study demonstrates that a concise communication skills training program based on the patient-centered care (PCC) model significantly enhances physicians' readiness for patient-centered interactions and improves patient satisfaction. The training's effectiveness across diverse demographics and specialties underscores its potential as a versatile tool for continuing medical education in Egypt's multispecialty settings. The notable improvement in patient satisfaction, especially among those with lower baseline PPOS scores, highlights the value of targeted educational interventions. While promising, further research with larger samples and longer follow-up is needed to assess the sustainability and broader applicability of these improvements in various healthcare environments.

Availability of data and material

The datasets during and/or analyzed during the current study available from the corresponding author on reasonable request.

## Authors consent of publication

All authors have read and approved the final manuscript and agreed on its submission to (International Journal of Medical Informatics).

## CRediT authorship contribution statement

**Mahmoud M. Samir:** Writing – review & editing, Writing – original draft, Conceptualization. **Nehal Mohamed Eisa:** Writing – review & editing, Supervision. **Heba Khaled:** Methodology, Formal analysis. **Wajid Syed:** Project administration, Funding acquisition. **Mahmood Basil A. Al-Rawi:** Project administration, Funding acquisition. **Ahmed Essam Abou Warda:** Validation, Project administration. **Nourhan M. Kamal:** Resources. **Reem S. Mahmoud:** Resources. **Abdelrahman SH. Refaee:** Visualization, Supervision.

## Funding

This study was supported by the Research Supporting Project, King Saud University, Saudi Arabia, (RSP2025R378) which provided funding for this work.

## Declaration of competing interest

The authors declare that they have no known competing financial interests or personal relationships that could have appeared to influence the work reported in this paper.

## Data Availability

The data that has been used is confidential.
